# Dialysis and Acid–Base Balance: A Comparative Physiological Analysis of Boston and Stewart Models

**DOI:** 10.3390/jcm14228206

**Published:** 2025-11-19

**Authors:** Nikolaos Kroustalakis, Eleftheria Maragkaki, Ariadni Androvitsanea, Ioannis Petrakis, Eleni Drosataki, Kleio Dermitzaki, Christos Pleros, Andreas Antonakis, Dimitra Lygerou, Eumorfia Kondili, Dimitris Georgopoulos, Kostas Stylianou

**Affiliations:** 1 Department of Nephrology, University General Hospital of Heraklion, 71500 Heraklion, Greece; nkroustalakis@yahoo.gr (N.K.); ariaandrovitsanea@gmail.com (A.A.); petrakgia@gmail.com (I.P.); ekderm@gmail.com (K.D.); xpleros@gmail.com (C.P.); antonakisandreas@yahoo.gr (A.A.); dimitra.ligerou@gmail.com (D.L.); 2 School of Medicine, Voutes, University of Crete, 70013 Heraklion, Greece; kondylie@uoc.gr (E.K.); georgopd@uoc.gr (D.G.); 3 Mesogeios Dialysis Center, Kalessa, 71500 Heraklion, Greece; elemaragleo@gmail.com; 4 Intensive Care Unit, University General Hospital of Heraklion, 71500 Heraklion, Greece

**Keywords:** hemodialysis, peritoneal dialysis, acid–base, Henderson–Hasselbalch, Stewart approach, strong ion difference, anion gap, bicarbonate, SIG

## Abstract

**Background:** The relative merits of the Henderson–Hasselbalch (HH) versus Stewart frameworks for interpreting dialysis-associated acid–base shifts remain debated. Dialysis alters systemic pH through exogenous bicarbonate delivery, chloride displacement, and removal of organic anions. We compared these approaches across hemodialysis (HD) and peritoneal dialysis (PD). **Methods:** We studied 53 HD patients with paired pre/post-HD blood gas and chemistry (106 observations) and 41 PD patients cross-sectionally, totaling 147 datasets. Derived variables followed the Figge/Stewart implementation [apparent SID (SIDa), effective SID (SIDe), strong ion gap (SIG), albumin-corrected anion gap (AGc)]. For HD, changes in pH (ΔpH) were modeled using HH predictors (ΔHCO_3_^−^, ΔPCO_2_) and Stewart predictors (ΔSIDa, ΔATOT, ΔPCO_2_). For cross-sectional data (pre-HD, post-HD, and PD), HH- and Stewart-based level models were fitted. Stewart-predicted pH was also computed using the Figge and the simplified Constable electroneutrality equation. **Results:** HD increased pH by 0.11, driven by ΔHCO_3_^−^ = +5.7 mΕq/L, ΔCl^−^ = −2.3 mEq/L, and declines in unmeasured anions (ΔSIG = −3.9; ΔAGc = −3.3). SIDa increased only marginally (+1.3 mEq/L), whereas SIDe rose by +5.3 mEq/L and fully tracked the alkalinization. In Δ-models, HH explained 90% of variance in ΔpH (R^2^ = 0.903) compared with 51% for Stewart (R^2^ = 0.514). In level models, HH explained 96% of pH variance versus 36% for Stewart. Bland–Altman analysis showed systematic overestimation of pH by the Figge and Constable approach (bias + 0.111), most pronounced pre-HD. PD patients had consistently higher AGc and SIG values than HD patients, indicating a greater burden of unmeasured anions. **Conclusions:** Alkalinization during HD is primarily attributable to bicarbonate gain, chloride displacement, and organic-anion clearance. The HH framework provides superior predictive performance for ΔpH, while closed-system Stewart formulations based on SIDa underestimate alkalinization. However, a broader physicochemical interpretation using SIDe and SIG, which incorporate bicarbonate and unmeasured anions, coherently describes the observed physiology. Future applications of the Stewart approach in dialysis should emphasize SIDe and SIG to better reflect the open-system physiology of both HD and PD. Our findings suggest that the HH model remains more predictive of alkalinization, while SIDe and SIG refine the physicochemical understanding.

## 1. Introduction

Acid–base changes during dialysis can be interpreted through two complementary frameworks. The traditional Henderson–Hasselbalch (HH) model relates pH to the ratio of bicarbonate (HCO_3_^−^) and carbon dioxide tension (PCO_2_) and has long underpinned clinical reasoning, treatment of acid base disorders, and dialysate prescriptions. In contrast, Stewart’s physicochemical approach [1] treats HCO_3_^−^ as a dependent variable and posits three independent determinants of pH: the strong ion difference (SID), PCO_2_, and the total concentration of non-volatile weak acids (ATOT). Abnormality of one or more of the independent variables underlies all acid–base disturbances [2]. Adjustment of the independent variables is the essence of all therapeutic interventions, because none of the “dependent variables” [e.g., pH, Base Excess (BE), HCO_3_^−^] can be changed primarily or individually. Instead, the dependent variables change, all of them simultaneously, if, and only if, one or more of the independent variables change [3]. In this view, pH changes arise from shifts in strong ions and weak acids rather than from “adding bicarbonate” per se.

Several studies have applied the Stewart framework in dialysis and critical illness. Libório et al. showed that in maintenance HD, acidosis was mainly attributable to unmeasured anions, hyperchloremia, and hyperphosphatemia, with chloride and unidentified anions accounting for nearly 90% of the metabolic component [4]. Prior comparative work has reported ~28% disagreement between Stewart-based and traditional classifications when the anion gap is left uncorrected for albumin. Lee et al. observed that in HD patients with metabolic acidosis, the strong ion gap (SIG) was consistently elevated and better captured unmeasured anions than conventional AG [5]. However, correcting the anion gap for albumin (AGc) materially improves its performance, and some studies show comparable discrimination between AGc and SIG. Moreover, there is no consistent evidence that Stewart-derived indices outperform traditional metrics in predicting clinical outcomes (e.g., mortality) [6].

In critically ill dialysis patients, chloride balance was highlighted as a key determinant of acidosis correction, with the relationship between plasma and dialysate chloride influencing the degree of improvement [7]. Constable and Kurtz et al. emphasized that while the Stewart approach is chemically rigorous, it does not surpass the Henderson–Hasselbalch framework in predictive accuracy—particularly in open systems like hemodialysis, where bicarbonate flux is the dominant driver [8,9].

Stewart theory predicts that the alkalinizing effect is mediated primarily by the sodium cation, which raises SID, while the administered HCO_3_^−^ equilibrates with CO_2_ and water, with excess CO_2_ subsequently eliminated. Thus, the rise in pH reflects a change in strong-ion composition, not an independent action of bicarbonate. This distinction is directly relevant to dialysis, where patients are exposed to large bicarbonate fluxes and dialysate chloride is simultaneously lower; both processes can shift SID and ATOT, while PCO_2_ often changes little across a session.

Despite several physiochemical analyses in dialysis, no prior study has directly evaluated, in the same cohort, how well the Stewart model predicts both absolute pH and dialysis-induced ΔpH compared with the Henderson–Hasselbalch framework, nor whether Figge/Constable-based pH predictions systematically deviate from measured values. This gap leaves uncertainty about the practical performance of the Stewart model under real-world bicarbonate-based dialysis conditions, which our analysis addresses through paired HD data, cross-modal comparisons, and Bland–Altman evaluation.

Accordingly, we designed two complementary analyses. First, a paired Δ-analysis in hemodialysis (HD) examined whether ΔpH was explained more accurately by the Henderson–Hasselbalch (HH) framework (ΔHCO_3_^−^, ΔPCO_2_) or by a Stewart model (ΔSIDa, ΔATOT, ΔPCO_2_).

Second, a cross-sectional level analysis pooled pre-HD, post-HD, and peritoneal dialysis (PD) observations to compare HH-based models against a Stewart model for predicting absolute pH.

In parallel, we decomposed the biochemical drivers of alkalinization, quantifying the specific contributions of chloride shifts (reflected in SIDa), changes in weak acids (ATOT; albumin and phosphate), and the burden of unmeasured anions (AGc/SIG), in the context of relatively stable PCO_2_ during dialysis.

## 2. Results

The study cohort comprised 94 patients, including 54 males (mean age 66.6 ± 12.2 years) and 40 females (mean age 68.6 ± 10.5 years), with an overall mean age of 67.5 ± 11.5 years. ANOVA showed no significant difference in age between males and females (F = 0.704, *p* = 0.404). Patients on PD (n = 41, mean age 67.8 ± 12.5 years) and HD (n = 53, mean age 67.2 ± 10.8 years) also had comparable ages, with no significant difference by dialysis modality (F = 0.062, *p* = 0.804). The distribution of dialysis modality was balanced across gender groups (42.6% of males and 45.0% of females on PD), and Chi-Square analysis confirmed the absence of association between sex and dialysis modality (χ^2^ = 0.054, *p* = 0.816). No significant differences were observed between males and females in biochemical or acid–base parameters, indicating that sex did not influence systemic electrolyte balance or acid–base homeostasis in this cohort (Supplementary Table S1).

### 2.1. Analysis of the Whole Study Group

Across the three groups (PD, pre-HD, post-HD), acid–base parameters and several electrolytes differed significantly (Table 1). The lowest pH was observed pre-HD (7.31), accompanied by reduced bicarbonate (~19 mmol/L), whereas post-HD showed clear alkalinization (pH 7.42, HCO_3_^−^ ~25 mmol/L; both ANOVA *p* < 0.001). PD patients exhibited intermediate values (pH 7.39, HCO_3_^−^ 23 mmol/L). PCO_2_ remained unchanged across groups (~40 mmHg, *p* = 0.94).

During HD, SIDa rose slightly (+1.39 mmol/L, *p* = 0.025) and ATOT remained stable (–0.40 mmol/L, *p* = 0.44), changes insufficient to explain the pH shift. As expected, potassium was higher pre-dialysis and decreased significantly after HD (*p* < 0.01). Chloride was lower in PD than pre-HD and declined modestly post-HD, while phosphate decreased sharply after HD. Albumin increased after HD, consistent with hemoconcentration.

Post hoc (Tukey) testing showed that pre-HD values differed significantly from both PD and post-HD for most parameters (pH, HCO_3_^−^, SIDe, SIG, AGc). Post-HD values converged toward PD for pH and bicarbonate but remained distinct in terms of lower SIG/AGc, indicating a greater burden of unmeasured anions in PD (Supplementary Tables S2 and S3).

#### 2.1.1. Regression Analysis of Absolute Measured pH Values Across Modalities

In the cross-sectional level analysis (all observations pooled, n = 147), the Stewart model using SIDa, ATOT, and PCO_2_ explained only 36% of the variance in measured pH (R^2^ = 0.362, adjusted R^2^ = 0.349; SEE = 0.061). In contrast, the Henderson–Hasselbalch framework, incorporating HCO_3_^−^ and PCO_2_, accounted for 96% of the variance (R^2^ = 0.961, adjusted R^2^ = 0.960; SEE = 0.015).

#### 2.1.2. Bland–Altman Analysis of Stewart-Predicted pH

We applied the Figge electroneutrality equation [5] to predict pH from SIDa, ATOT and PCO_2_ in the whole group and the subgroups.

**Figure 1:**
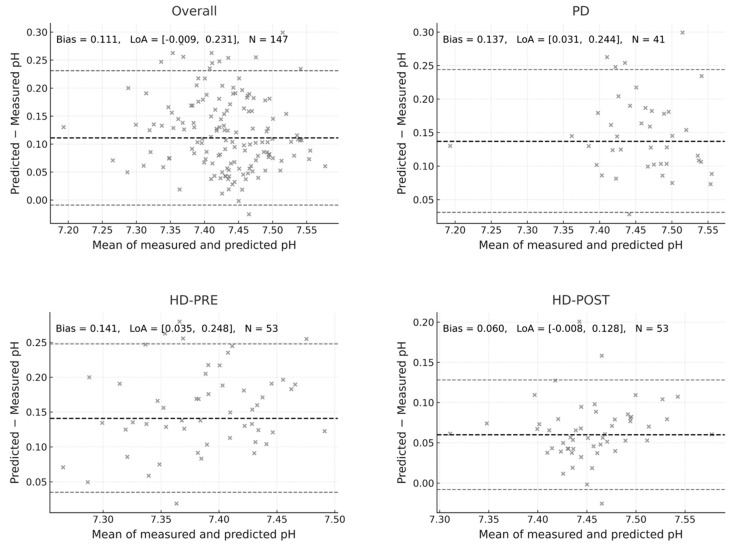
Bland–Altman analysis of measured versus predicted pH using the Figge-Fencl equation.

We found that it systematically overestimated the measured pH across all modalities, as is shown with the Bland–Altman analysis below. In the overall cohort (n = 147), the mean bias was +0.111 pH units. In PD patients the bias was +0.137; pre-HD, the bias was +0.141 and the post-HD group the bias was +0.06 (Figure 1).

Thus, the Figge approach consistently predicted higher pH values than measured, with the largest overestimation observed before HD, slightly less in PD, and the lowest in post-dialysis samples. Importantly, in all subgroups the 95% limits of agreement remained entirely positive, indicating a systematic upward bias. For sensitivity analysis we also applied the simplified Constable equation [6], which produced a slightly higher systematic bias (+0.112 pH units), indicating that both Stewart-based formulations overestimate pH values.

Each panel depicts the agreement between measured arterial pH and pH predicted using the Figge–Fencl Stewart-based formulation in all patients (Overall) and in the subgroups of peritoneal dialysis (PD), hemodialysis before treatment (HD-PRE), and after treatment (HD-POST). The thick dashed line indicates the mean bias, and the thin dashed lines represent the 95% limits of agreement (LoA = bias ± 1.96 SD). Numerical annotations show the bias, LoA range, and sample size (N) for each comparison. The overall bias was +0.111 pH units (LoA −0.009 to +0.231; N = 147), higher in HD-PRE (+0.141) and PD (+0.137), and lower in HD-POST (+0.060). A positive bias indicates systematic overestimation of alkalinization by the Figge–Fencl equation relative to measured values.

#### 2.1.3. Strong Ion Gap or Corrected Anion Gap

**Figure 2:**
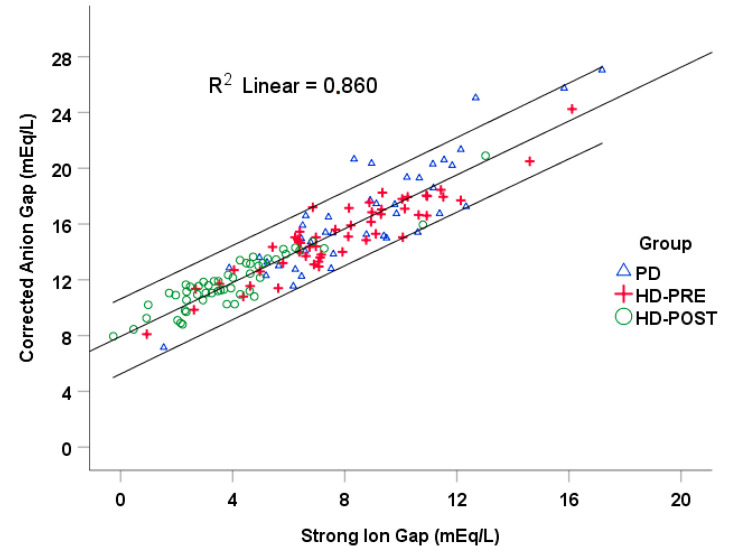
Relationship between albumin-corrected anion gap (AGc, *Y* axis) and strong-ion gap (SIG, *X* axis). Scatterplot illustrating the association between AGc and SIG across all dialysis samples. The solid line represents the linear regression fit, while the outer shaded bands depict the 95% prediction interval (PI), capturing the expected range of individual observations around the fitted line. A strong positive correlation was observed between AGc and SIG (AGc = 7.94 + 0.966·SIG; R^2^ = 0.86, *p* < 0.0001), indicating that both indices provide comparable estimates of the unmeasured anion burden. Symbols denote patient subgroups: peritoneal dialysis (PD, blue triangles, N = 47), hemodialysis before treatment (HD-PRE, red crosses, N = 53), and after treatment (HD-POST, green circles, N = 53). Units expressed in milliequivalents per liter (mEq/L).

In the regression analysis we observed a strong linear relationship between AGc and SIG (AGc = 7.94 + 0.97·SIG (R^2^ = 0.86, *p* < 0.0001, N = 147)) (Figure 2). The slope was close to 1.0, indicating parallel changes in the two variables across the cohort. The intercept was approximately 8 mEq/L.

### 2.2. Analysis of Changes in the HD Group (Pre/Post)

Beyond cross-sectional comparisons, we analyzed paired pre–post changes within the hemodialysis (HD) cohort to identify which modeling framework best describes ΔpH. Post-HD, pH increased by 0.11, base excess rose by 7.04, and bicarbonate increased by 5.67, consistent with effective alkali delivery (Table 2). In contrast, SIDa changed only marginally (by +1.39) because opposing shifts largely canceled each other out: chloride declined (which tends to raise SIDa) while potassium also declined substantially (which tends to lower SIDa). ATOT remained relatively stable, reflecting a compensatory pattern of increased albumin (hemoconcentration) and decreased phosphate (dialytic clearance). As expected, PCO_2_ showed no significant net change during HD, consistent with steady CO_2_ content in the dialysate. Ordinary least-squares regression on paired ΔpH (Table 3) favored the Henderson framework (ΔHCO_3_ + ΔPCO_2_, R^2^ = 0.903, adj. R^2^ = 0.899), while the Stewart model (ΔSIDa, ΔATOT, ΔPCO_2_) explained about half of the variance (R^2^ = 0.514, adj. R^2^ = 0.485).

Regarding the three linear models for ΔpH, the analysis clearly favors the Henderson model (ΔHCO_3_^−^ + ΔPCO_2_), which demonstrated the highest R^2^/adjusted R^2^, the lowest SEE, and the most favorable AIC/BIC values, indicating superior fit and efficiency. The ΔHCO_3_-only model underfits (low R^2^, higher SEE, worse AIC/BIC), confirming that bicarbonate alone does not capture inter-patient variability in ΔpH. The Stewart-only model performs moderately (R^2^ = 0.485) but is decisively outperformed by Henderson on all criteria.

Coefficient patterns are physiologically coherent. In the Henderson model, ΔHCO_3_ is strongly positive and ΔPCO_2_ strongly negative—both highly significant—consistent with the Henderson–Hasselbalch framework. In the Stewart model, ΔSIDa is a positive predictor (significant), ΔPCO_2_ remains negative (significant), and ΔATOT is non-significant—compatible with the small, opposing albumin/phosphate changes within a single HD session.

In this cohort, mean ΔSIDa was +1.39 mmol/L, whereas ΔHCO_3_ averaged +5.67 mmol/L with ΔPCO_2_ +0.38 mmHg. Using the fitted coefficients, the Stewart slope for ΔSIDa (0.009 pH units per mmol/L) implies an expected ΔpH of only ~0.012—far below the observed effect. By contrast, the Henderson model coefficients (0.0233 for ΔHCO_3_ and −0.011 for ΔPCO_2_) yield a predicted ΔpH ≈ 0.126 for ΔHCO_3_ = +5.6 mmol/L and ΔPCO_2_ = +0.38 mmHg, closely matching the observed mean ΔpH of +0.116.

## 3. Discussion

In this cohort of 41 PD patients and 53 HD patients, we analyzed 147 blood gas samples to compare the explanatory performance of the “Boston” and the Stewart frameworks across modalities and within paired HD sessions. Hemodialysis produced a characteristic alkalinization with substantial increase in HCO_3_^−^ accompanied by a fall in chloride and a reduction in unmeasured anions, yielding a mean ∆pH of ~0.11.

Importantly, ATOT remained essentially unchanged across dialysis because hemoconcentration-related increases in albumin were offset by dialytic phosphate removal. Consequently, changes in weak-acid charge cannot account for the rise in pH.

The mean SIDa increased modestly by +1.39 mmol/L—far too little to explain the magnitude of the pH or bicarbonate change. Mechanistically, sodium remained relatively stable during HD (+0.43), while the decrease in chloride (ΔCl^−^ = −2.34) was partially neutralized in the SIDa equation by a concomitant fall in potassium (−1.53); these opposing charge shifts blunted the net change in SIDa. The fall in chloride and potassium is consistent with the dialysate composition with higher HCO_3_^−^ and lower Cl^−^ and K^+^ than plasma.

Moreover, PCO_2_ in blood mirrored the near-constant CO_2_ content maintained in the dialysate, so neither ATOT nor PCO_2_ shifted in a way that could drive the observed alkalinization within a strictly Stewart formulation.

To probe the unmeasured-anion component, we examined SIG and AGc in the whole group. According to our data the relationship between the two of them was AGc = 8 + 0.97·SIG (Figure 2). In his paper in 1999, Constant supports that SIG is more specific in detecting the presence of unmeasured strong ions in plasma than the anion gap [8]. Similar results were reported by Lee et al. [5]. In our analysis AGc and SIG track the same physiology—unmeasured anions—but on slightly different zero points. The perfect slope (~1) indicates they move together almost identically, while the ~8 mEq/L intercept reflects definitional choices (albumin/phosphate/organic anions for AGc vs. organic anions in SIG). Practically, AGc and SIG are interchangeable proxies for the unmeasured-anion burden. While SIDa rose only slightly, both SIG and AGc declined substantially, indicating clearance of unmeasured anions. This is consistent with removal of uremic organic acids—e.g., indoxyl sulfate, hippurate, p-cresyl sulfate, urate, ketoacids.

From a Stewart perspective, the pH rise is captured by a pronounced increase in SIDe and a fall in SIG; critically, the SIDe increase is driven almost entirely by the bicarbonate gain. In parallel, the relatively low chloride in the dialysate (due to displacement by the bicarbonate load) corrects the hyperchloremic fraction of acidosis, further contributing to the net alkalinization.

From the Henderson–Hasselbalch viewpoint, the interpretation is straightforward: exogenous bicarbonate enters from the dialysate and pH increases with PaCO_2_ essentially unchanged. Our paired analysis showed that the observed rise in pH was almost completely explained by the increase in plasma bicarbonate.

Both frameworks therefore describe the same physical process from different angles: the dialysate is relatively rich in HCO_3_^−^, poor in Cl^−^, and effectively devoid of organic anions. The small acetate/citrate load is titrated by bicarbonate to yield a stable CO_2_ concentration in the effluent, while the corresponding anions are cleared. Thus, HCO_3_^−^ accumulation, Cl^−^ displacement, and organic-anion removal occur simultaneously. This explains why SIDa—which excludes HCO_3_^−^—underestimates the alkalinizing effect, whereas SIDe and SIG, which include HCO_3_^−^, track the process more faithfully.

A conceptual paradox emerges here. In Stewart’s approach, bicarbonate is a dependent variable, yet in practice the model requires HCO_3_^−^ (via SIDe) to reveal the fall in SIG. Without the bicarbonate term, the physicochemical model would miss the contribution of organic-anion removal—an effect that, conversely, is readily detected by the cAG. Thus, while Stewart’s approach elegantly emphasizes independent strong ions and weak acids, its practical application in HD paradoxically leans on bicarbonate to uncover the exact changes in SIG.

In its conventional closed-system form (using only SIDa, ATOT, and PCO_2_ as independent variables), the Stewart model underestimated the alkalinization observed during HD, because these variables changed very little. In contrast, when the analysis was extended to an open-system context that also considers the exogenous bicarbonate load (through SIDe), the rise in pH was fully captured. At the same time, the fall in SIG/AGc reflected removal of unmeasured organic anions. Thus, using SIDe and SIG rather than SIDa alone allows the Stewart framework to describe the alkalinizing effect of dialysis, showing that the apparent mismatch comes from the restricted closed-system implementation, which cannot account for bicarbonate infusion. These findings also align with prior critiques of the closed-system Stewart formulation. Constable emphasized that SIDa cannot capture alkalinization when bicarbonate is added or removed and that SIDe/SIG—because they incorporate HCO_3_^−^ and weak-acid charge—provide a more complete representation of pH under such conditions. Similarly, Rastegar noted that in clinical settings where changes in bicarbonate dominate the acid–base response, the Henderson–Hasselbalch framework remains more predictive than a Stewart model restricted to SIDa, ATOT and PCO_2_. Our results in dialysis, an archetypal open system with substantial bicarbonate gain and organic-anion clearance, are fully consistent with these observations.

With our analysis we also found that the Figge–Constable equations systematically overestimated measured pH in the overall cohort, most notably pre-HD, with smaller bias post-HD. If the problem were primarily due to differences in ion measurement techniques that have changed since the time of Figge and Constable, the bias would have been consistent pre- and post-dialysis, since sodium and chloride were measured using the same contemporary methods on the same analyzer. The reduction in bias after dialysis, when mean pH increased from 7.31 to 7.42, suggests that methodological factors cannot explain the mismatch. Rather, the shift toward alkalemia likely altered the effective dissociation behavior of albumin and phosphate, bringing their apparent K_a_ values closer to those assumed by the Figge–Constable model. In addition, dialysis reduces the burden of organic anions, thereby decreasing the requirement for counterbalancing strong cations and simplifying the ionic milieu in a way that further enhances model performance. Taken together, these findings suggest that the choice of dissociation constants may contribute substantially to prediction error, and that reevaluating the K_a_ values under the dynamic pH and organic anion conditions encountered during hemodialysis may improve the accuracy of the Stewart approach.

Our findings are consistent with those of Hernández et al., who, in a cohort of 35 HD patients, found that the Henderson-based framework provided a closer fit to measured pH [10]. Consistent results were also observed in the intensive care setting: in a study of 172 patients, the standard base excess (SBE) model was a superior predictor of acidemia and alkalemia compared with the apparent SID-based model [11], while in another ICU cohort the bicarbonate–cAG approach showed diagnostic performance comparable to that of the Stewart framework [12].

Although our study focuses on chronic dialysis, broader evidence suggests that SIG may have clinical relevance beyond dialysis. In patients with AKI and metabolic acidosis, SIG has been independently associated with both short- and long-term mortality [13], underscoring its prognostic value and supporting its use in general nephrology practice.

With regard to the PD group, patients exhibited higher AGc and SIG values than HD patients, indicating a greater burden of unmeasured anions in PD. This observation aligns with the findings of Klabosh et al., who demonstrated that metabolic acidosis in PD is characterized by an increased SIG compared with healthy controls [14].

This study has several strengths. First, it uniquely combines paired pre/post hemodialysis data with a peritoneal dialysis cohort, allowing direct comparison of acid–base responses across modalities. Second, it applies both Henderson–Hasselbalch and Stewart–Figge frameworks in parallel, not only at absolute levels but also in Δ-models, providing a rigorous head-to-head assessment of predictive performance. Finally, the dataset is clinically relevant, reflecting routine dialysis practice with contemporary bicarbonate-based solutions, and the results directly inform the ongoing debate over the utility of physicochemical versus traditional models in real-world dialysis physiology.

Limitations include the single-cohort design and modest sample size. Dialysate composition (HCO_3_^−^/Cl^−^) was uniform rather than randomized, and we did not directly quantify the full panel of organic anions. Additionally, the generalizability of our findings may be influenced by inter-center variability in dialysate composition. Differences in bicarbonate concentration, chloride content, potassium bath, or acetate/citrate buffering can modify the relative contributions of HCO_3_^−^ gain, chloride displacement, and organic-anion clearance to pH changes; thus, the exact magnitude of the effects observed here may differ in dialysis units using alternative prescriptions. Nevertheless, the conceptual distinction between closed- and open-system Stewart interpretation, and the superior predictive performance of the Henderson–Hasselbalch framework under bicarbonate-based dialysis, should remain broadly applicable

## 4. Materials and Methods

### 4.1. Study Population, Dialysis Solutions and Sampling

We enrolled 53 chronic hemodialysis (HD) patients with paired arterial blood gases and blood chemistry immediately before and after routine sessions (106 observations total) and 41 stable peritoneal dialysis (PD) patients sampled cross-sectionally (41 observations). Accordingly, cross-sectional (“level”) analyses comprised 147 observations (pre-HD, post-HD, PD).

Dialysis treatments were performed with a Fresenius 4008S machine (Fresenius Medical Care, Bad Homburg, Germany). The standard dialysate composition was sodium 138 mEq/L, potassium 2.0 mEq/L, calcium 3.0 mEq/L, magnesium 1.0 mEq/L, chloride 108 mEq/L, acetate 2.7 mEq/L, and bicarbonate 32 mEq/L, with minor adjustments according to individual clinical requirements.

Peritoneal dialysis was performed with bicarbonate-buffered bicaVera solutions (Fresenius Medical Care, Bad Homburg, Germany) and, when indicated, with icodextrin (Extraneal, Baxter Healthcare, Deerfield, IL, USA) for long dwell exchanges. After mixing of the dual chambers, the final composition of bicaVera was sodium 134 mEq/L, calcium 1.75 mmol/L, magnesium 0.5 mmol/L, chloride 101 mEq/L, bicarbonate 34 mEq/L, and glucose at 1.5%, 2.3%, or 4.25% (corresponding to 83, 126, or 236 mmol/L). Icodextrin solution contained sodium 133 mEq/L, calcium 1.75 mmol/L, magnesium 0.25 mmol/L, chloride 96 mEq/L, lactate 40 mEq/L, and 7.5% icodextrin as the osmotic agent.

Patients continued on their individualized PD and HD prescriptions without modification on the day of measurement. The study was conducted in accordance with the principles of the Declaration of Helsinki. All participants provided written informed consent prior to enrollment. As all laboratory tests were part of routine clinical care and no additional interventions were performed, the requirement for formal ethics committee approval was waived by the local institutional review board.

### 4.2. Measurements and Derived Variables

Measured variables included pH, PCO_2_ (mmHg), HCO_3_^−^ (mEq/L), Na^+^, K^+^, ionized Ca^2+^ (iCa), ionized Mg^2+^ (iMg), Cl^−^ (all in mEq/L), lactate (mmol/L), albumin (g/L), and phosphate (P) (mmol/L).

We implemented the Figge/Stewart framework [2,3,8,15,16]. The equations used were defined as follows:

Weak acid anions:

ATOT = [A^−^] = [Alb^−^] + [Pi^−^] (1)

where [Alb^−^] is albumin charges and [Pi^−^] is phosphate charges; [Alb^−^] = [Alb]·(0.123·pH − 0.631) and [P^−^] = [P]·(0.309·pH − 0.469), with [Alb] in g/L and [P] in mmol/L.

Apparent strong-ion difference:

SIDa = ([Na^+^] + [K^+^] + [iCa^2+^] + [iMg^2+^]) − ([Cl^−^] + [lactate]) (2)

Effective strong-ion difference:

SIDe = [HCO_3_^−^] + ATOT (3)

Strong-ion gap:

SIG = SIDa − SIDe(4)

Stewart-predicted pH (Figge electroneutrality equation):

SIDa = S·PCO_2_·10^(pH − pK_a_) + [Alb]·(0.123·pH − 0.631) + [P]·(0.309·pH − 0.469) (5)

S = 0.0301 mmol/L/mmHg and pK_a_ = 6.10.

The Constable simplified equation, which is derived from Equation (5), was solved numerically for each sample to obtain the Constable-predicted pH:

pH = log_10_ (2 [SID^+^]/(K′_1_·S·PCO_2_ + K_a_([ATOT] − [SID^+^]) + √((K′_1_·S·PCO_2_ + K_a_([SID^+^] + [ATOT]))^2^ − 4K_a_^2^ [SID^+^][ATOT]))) (6)

where K′_l_ is the apparent equilibrium constant for the Henderson–Hasselbalch equation, S is the solubility of CO_2_ in plasma, PCO_2_ is arterial carbon dioxide tension, K_a_ is the dissociation constant for weak acids, [ATOT] is the total concentration of weak acids and [SID^+^] is the strong-ion difference SIDa.

Anion gap was calculated as

AG = [Na^+^] − ([Cl^−^] + [HCO_3_^−^]) (7)

and corrected for the effect of abnormal albumin concentration with the formula

AGc = AGobserved + 0.25 × ([normal Alb] − [observed Alb])

### 4.3. Statistical Analysis

For paired HD Δ-models, the outcome was ΔpH (post − pre). We fitted a Henderson–Hasselbalch model (ΔpH defined by ΔHCO_3_^−^ and ΔPCO_2_) and a Stewart model (ΔpH defined by ΔSIDa, ΔATOT and ΔPCO_2_).

For cross-sectional level models (all observations; n = 147), the outcome was pH, modeled as HH model (pH defined by HCO_3_^−^ and PCO_2_) and Stewart model (pH defined by SIDa, ATOT and PCO_2_). All models used ordinary least squares, and prespecified performance metrics were R^2^/adjusted R^2^.

All statistical analyses were performed using IBM SPSS Statistics, version 26 (IBM Corp., Armonk, NY, USA). As SPSS does not provide a built-in function for Bland–Altman analysis, we generated the corresponding plots using R code version 4.3.2 (R Foundation for Statistical Computing, Vienna, Austria) obtained with the assistance of ChatGPT (OpenAI GPT-5.1 model; OpenAI, San Francisco, CA, USA).

Model assumptions for linear regression were systematically assessed. Normality of ΔpH and model residuals was verified using Shapiro–Wilk tests (*p* = 0.87) and Q–Q plot inspection, confirming an approximately normal distribution without need for transformation. Homoscedasticity and independence of residuals were evaluated graphically and showed no violations. Multicollinearity was minimal (VIF < 2 for all predictors). Influential observations were assessed with Cook’s distance; although two data points exceeded the conventional 4/n threshold, they reflected physiologically plausible changes and were retained. No outliers were removed.

## 5. Conclusions

The Stewart model struggles to account for HD-induced alkalinization when applied in its closed-system form, as it does not natively accommodate exogenous bicarbonate delivery; thus, despite sizeable increases in pH and HCO_3_^−^, its independent variables (SIDa, ATOT, PCO_2_) changed little. By contrast, the “Boston” framework accurately predicted the observed ∆pH from ∆HCO_3_^−^ and ∆PCO_2_, reflecting the clinical reality that bicarbonate flux from dialysate is the dominant driver. Nevertheless, a broader physicochemical interpretation using SIDe and SIG—which incorporate bicarbonate and capture clearance of unmeasured anions—coherently described the alkalinizing effect. The apparent discrepancy therefore lies not in the Stewart framework itself but in its restricted implementation. In the context of both hemodialysis and peritoneal dialysis, future applications of the Stewart framework should rely on SIDe and SIG, which incorporate bicarbonate and unmeasured anion clearance, to more faithfully represent the open-system physiology of dialysis.

## Figures and Tables

**Table 1:** Descriptive analysis of the whole group. Values are mean ± SD. Group sample sizes are shown in column headers. ANOVA *p*-value refers to the overall test across PD, pre-HD, and post-HD.

Parameter	PD (n = 41)	Pre-HD (n = 53)	Post-HD (n = 53)	ANOVA p
**Na**	136.83 ± 4.61	137.06 ± 2.55	137.49 ± 1.79	0.5640
**K**	4.57 ± 0.99	5.20 ± 0.75	3.67 ± 0.41	<0.0001
**iCa**	2.17 ± 0.30	2.38 ± 0.42	2.59 ± 0.30	<0.0001
**iMg**	1.33 ± 0.32	1.33 ± 0.23	1.14 ± 0.10	<0.0001
**Cl**	98.80 ± 6.57	102.57 ± 3.19	100.23 ± 2.04	<0.0001
**Lactate**	1.77 ± 0.77	2.38 ± 0.80	2.26 ± 0.77	0.0007
**Alb**	33.10 ± 5.97	39.51 ± 3.59	41.25 ± 4.67	<0.0001
**P**	1.84 ± 0.81	1.78 ± 0.50	0.96 ± 0.28	<0.0001
**pH**	7.38 ± 0.07	7.31 ± 0.05	7.42 ± 0.05	<0.0001
**HCO_3_^−^**	23.13 ± 4.12	19.44 ± 2.59	25.11 ± 1.40	<0.0001
**SIDa**	44.34 ± 3.64	41.03 ± 2.54	42.41 ± 1.96	<0.0001
**ATOT**	12.50 ± 2.12	13.77 ± 1.42	13.37 ± 1.56	0.0018
**SIDe**	35.63 ± 3.44	33.21 ± 2.72	38.48 ± 1.64	<0.0001
**SIG**	8.70 ± 3.10	7.82 ± 2.88	3.94 ± 2.27	<0.0001
**PCO_2_**	39.48 ± 7.82	39.45 ± 5.72	39.83 ± 4.80	0.9416
**AG**	14.90 ± 4.11	15.05 ± 2.74	12.16 ± 2.31	<0.0001
**AGc**	16.62 ± 4.01	15.18 ± 2.78	11.85 ± 2.14	<0.0001
**BE**	−1.91 ± 4.34	−6.33 ± 2.78	0.71 ± 1.56	<0.0001

Note: Na^+^, K^+^, Cl^−^, lactate, ionized Ca^2+^, ionized Mg^2+^, SIDa, SIDe, SIG, AG, AGc and BE are expressed in mEq/L (strong-ion charge equivalents). Phosphate (P) is expressed in mmol/L, albumin in g/L, and PCO_2_ in mmHg; pH is dimensionless.

**Table 2:** Descriptive statistics for the HD group and mean change (pre → post). Values are mean ± SD; *p*-values are for paired pre–post differences.

Parameter	Pre-HD (n = 53) (Mean ± SD)	Post-HD (n = 53) (Mean ± SD)	Change Δ (Mean ± SD)	*p*-Value
**Na**	137.06 ± 2.55	137.49 ± 1.79	0.43 ± 2.26	0.1676
**K**	5.20 ± 0.75	3.67 ± 0.41	−1.53 ± 0.61	<0.0001
**iCa**	2.38 ± 0.42	2.59 ± 0.30	0.21 ± 0.45	0.0015
**iMg**	1.33 ± 0.23	1.14 ± 0.10	−0.19 ± 0.17	<0.0001
**Cl**	102.57 ± 3.19	100.23 ± 2.04	−2.34 ± 2.84	<0.0001
**LA**	2.38 ± 0.80	2.26 ± 0.77	−0.13 ± 1.00	0.3627
**Alb**	39.51 ± 3.59	41.25 ± 4.67	1.74 ± 3.06	0.0001
**P**	1.78 ± 0.50	0.96 ± 0.28	−0.82 ± 0.38	<0.0001
**pH**	7.31 ± 0.05	7.42 ± 0.05	0.11 ± 0.06	<0.0001
**HCO_3_**	19.44 ± 2.59	25.11 ± 1.40	5.67 ± 2.20	<0.0001
**SIDa**	41.03 ± 2.54	42.41 ± 1.96	1.39 ± 2.14	<0.0001
**ATOT**	13.77 ± 1.42	13.37 ± 1.56	−0.40 ± 1.04	0.0076
**SIDe**	33.21 ± 2.72	38.48 ± 1.64	5.27 ± 2.72	<0.0001
**SIG**	7.82 ± 2.88	3.94 ± 2.27	−3.88 ± 2.53	<0.0001
**PCO_2_**	39.45 ± 5.72	39.83 ± 4.80	0.38 ± 5.54	0.6236
**AG**	15.05 ± 2.74	12.16 ± 2.31	−2.89 ± 2.17	<0.0001
**AGc**	15.18 ± 2.78	11.85 ± 2.14	−3.33 ± 2.25	<0.0001
**BE**	−6.33 ± 2.78	0.71 ± 1.56	7.04 ± 2.65	<0.0001

Note: Ionized Ca^2+^ and Mg^2+^ values are automatically standardized to 37 °C by the blood gas analyzer.

**Table 3:** Ordinary Least Squares models on paired ΔpH (n = 53). Entries show B (SE); standardized β; *p*-value. AIC = Akaike Information Criterion; BIC = Bayesian Information Criterion; SEE = Standard Error of the Estimate (N = 53).

Variable/Metric	Stewart (ΔSIDa, ΔATOT, ΔPCO_2_)	Henderson (ΔHCO_3_ + ΔPCO_2_)	ΔHCO_3_-Only
**ΔSIDa**	0.009 (0.003); β = 0.324; *p* < 0.01		
**ΔATOT**	0.008 (0.006); β = 0.140; *p* = 0.214		
**ΔPCO_2_**	−0.006 (0.001); β = −0.580; *p* < 0.001	−0.011 (0.001); β = −1.005; *p* < 0.001	
**ΔHCO_3_**		0.023 (0.001); β = 0.851; *p* < 0.001	0.010 (0.004); β = 0.362; *p* < 0.01
**R^2^/Adjusted R^2^**	0.514/0.485	0.903/0.899	0.131/0.114
**SEE**	0.0432	0.0191	0.0566
**F (p)**	17.29 (*p* < 0.001)	231.87 (*p* < 0.001)	7.69 (*p* < 0.01)
**AIC/BIC**	−178.9/−171.0	−266.1/−260.2	−152.1/−148.1

## Data Availability

The raw data supporting the conclusions of this article will be made available by the authors on request.

## Supplementary Materials

The following supporting information can be downloaded at https://www.mdpi.com/article/10.3390/jcm14228206/s1: Table S1: Comparison of biochemical and acid–base parameters by gender (ANOVA); Table S2: Changes in hemodialysis patients; Table S3: Post hoc (Tukey HSD) comparisons among PD, HD-pre, and HD-post.
