# Supplementary material for: Dialysis and Acid–Base Balance: A Comparative Physiological Analysis of Boston and Stewart Models
Source: J Clin Med. 2025 Nov 19;14(22):8206. doi: 10.3390/jcm14228206 (PMC12653126; doi:10.3390/jcm14228206)
Supplement: Supplementary file 1 [file jcm-14-08206-s001.zip › jcm-3984434-supplementary.pdf]

Table S1. Comparison of Biochemical and Acid–Base Parameters by Gender (ANOVA)

Variable	Male (Mean ± SD) N=54	Female (Mean ± SD) N=40	Total (Mean ± SD) N=94	ANOVA p-value
**age**	66.61 ± 12.21	68.63 ± 10.47	67.47 ± 11.49	0.404
**Na**	137.37 ± 3.91	136.40 ± 3.04	136.96 ± 3.58	0.195
**K**	4.97 ± 0.93	4.88 ± 0.89	4.93 ± 0.91	0.632
**iCa**	2.32 ± 0.42	2.26 ± 0.33	2.29 ± 0.38	0.484
**iMg**	1.37 ± 0.27	1.29 ± 0.27	1.33 ± 0.27	0.183
**Cl**	101.57 ± 5.27	100.05 ± 5.21	100.93 ± 5.27	0.167
**Lactic**	2.05 ± 0.87	2.20 ± 0.81	2.12 ± 0.84	0.41
**Alb**	36.98 ± 5.84	36.35 ± 5.62	36.71 ± 5.72	0.6
**P**	1.86 ± 0.73	1.73 ± 0.52	1.81 ± 0.65	0.312
**pH**	7.33 ± 0.07	7.36 ± 0.08	7.34 ± 0.07	0.081
**HCO3**	20.76 ± 3.48	21.44 ± 4.20	21.05 ± 3.80	0.389
**SIDa**	42.39 ± 3.42	42.58 ± 3.57	42.47 ± 3.47	0.801
**Atot**	13.35 ± 1.87	13.03 ± 1.86	13.22 ± 1.86	0.409
**SIDe**	34.11 ± 3.31	34.47 ± 3.24	34.26 ± 3.27	0.595
**SIG**	8.28 ± 2.77	8.10 ± 3.30	8.21 ± 2.99	0.773
**PCO2**	40.12 ± 7.19	38.59 ± 5.89	39.47 ± 6.68	0.276
**AG**	15.04 ± 3.19	14.91 ± 3.68	14.98 ± 3.39	0.852
**AGc**	15.80 ± 3.45	15.82 ± 3.43	15.81 ± 3.43	0.973
**BE**	-4.75 ± 3.81	-3.99 ± 4.58	-4.43 ± 4.14	0.389
**Na_Cl**	35.80 ± 3.74	36.35 ± 3.79	36.03 ± 3.75	0.483

ANOVA analysis confirmed that no significant gender-related differences were present across biochemical or acid–base parameters (all $p > 0.05$).

Table S2. Changes in Hemodialysis Patients

The analysis of paired pre- and post-dialysis samples (paired t-test) demonstrated significant changes in most acid–base and electrolyte parameters (N=53). Differences (Δ = POST−PRE) are summarized below:

Variable	Δ (POST−PRE)	p
**AGc**	-3.328 ± 2.252	<0.0001
**Alb**	+1.736 ± 3.058	0.0001
**iCa**	+0.207 ± 0.451	0.002
**Cl**	-2.340 ± 2.842	<0.0001
**HCO3**	+5.668 ± 2.196	<0.0001
**K**	-1.530 ± 0.611	<0.0001
**LA**	-0.126 ± 1.002	0.36
**iMg**	-0.160 [0.160]	<0.0001
**Na**	+0.434 ± 2.258	0.17
**P**	-0.819 ± 0.383	<0.0001
**PCO2**	+0.375 ± 5.537	0.62
**SIDa**	+1.386 ± 2.138	<0.0001
**SIDe**	+5.270 ± 2.717	<0.0001
**SIG**	-3.884 ± 2.532	<0.0001
**pH**	+0.111 ± 0.060	<0.0001

Post-dialysis values showed a consistent rise in pH and bicarbonate, a fall in serum potassium, phosphate, corrected anion gap, and strong ion gap. Effective SIDe increased substantially, while apparent SIDa also rose but to a lesser extent. Sodium and PCO_2 did not change significantly.

Table S3. Post hoc (Tukey HSD) comparisons among PD, HD-pre, and HD-post. The numbers show the difference between two groups. The statistical significance is shown in parentheses; *ns: not significant*

Variable	PD vs HD-pre	PD vs HD-post	HD-pre vs HD-post
**Na**	-0.23 (p=0.9325 ns)	-0.66 (p=0.5553 ns)	-0.43 (p=0.7473 ns)
**K**	-0.63 (p=0.0002)	0.90 (p=0.0000)	1.53 (p=0.0000)
**iCa**	-0.21 (p=0.0119)	-0.42 (p=0.0000)	-0.21 (p=0.0071)
**iMg**	-0.00 (p=0.9991 ns)	0.19 (p=0.0003)	0.19 (p=0.0001)
**Cl**	-3.76 (p=0.0001)	-1.42 (p=0.2282 ns)	2.34 (p=0.0117)
**Lactate**	-0.61 (p=0.0007)	-0.49 (p=0.0088)	0.13 (p=0.6835 ns)
**Alb**	-6.41 (p=0.000)	-8.15 (p=0.0000)	-1.74 (p=0.1463 ns)
**P**	0.06 (p=0.8466 ns)	0.88 (p=0.0000)	0.82 (p=0.0000)
**pH**	0.08 (p=0.0000)	-0.04 (p=0.0085)	-0.11 (p=0.0000)
**HCO3**	3.69 (p=0.0000)	-1.98 (p=0.0026)	-5.67 (p=0.0000)
**SIDa**	3.31 (p=0.0000)	1.92 (p=0.0025)	-1.39 (p=0.0258)
**Atot**	-1.26 (p=0.0013)	-0.87 (p=0.0390)	0.40 (p=0.4477 ns)
**SIDe**	2.43 (p=0.0001)	-2.84 (p=0.0000)	-5.27 (p=0.0000)
**SIG**	0.88 (p=0.2728 ns)	4.77 (p=0.0000)	3.88 (p=0.0000)
**PCO2**	0.03 (p=0.9997 ns)	-0.35 (p=0.9599 ns)	-0.38 (p=0.9461 ns)
**AG**	-0.16 (p=0.9666 ns)	2.74 (p=0.0001)	2.89 (p=0.0000)
**AGc**	1.45 (p=0.0551 ns)	4.77 (p=0.0000)	3.33 (p=0.0000)
**BE**	4.42 (p=0.0000)	-2.63 (p=0.0001)	-7.04 (p=0.0000)
